# Using hospital-based studies of community-onset bloodstream infections to make inferences about typhoid fever incidence

**DOI:** 10.1111/tmi.13319

**Published:** 2019-12-21

**Authors:** Christian S. Marchello, Ariella P. Dale, Sruti Pisharody, John A. Crump

**Affiliations:** 1 Centre for International Health, University of Otago, Dunedin, New Zealand; 2 Colorado Department of Public Health and Environment, Denver, CO, USA; 3 Duke University School of Medicine, Durham, NC, USA

**Keywords:** *Salmonella* Typhi, typhoid fever, prevalence, modelling

## Abstract

**Objectives:**

Hospital-based studies of community-onset bloodstream infections (CO-BSI) are less resource-intensive to carry out than population-based incidence studies. We examined several metrics capturing the potential role of *Salmonella* Typhi as a cause of CO-BSI for making inferences about incidence.

**Methods:**

We systematically reviewed three databases for hospital-based studies of CO-BSI. We determined, by study, the prevalence and rank order of *Salmonella* among pathogenic bloodstream isolates, and the prevalence ratio of *Salmonella* Typhi to *Escherichia coli* (S:E ratio). We then describe these hospital-based study metrics in relation to population-based typhoid fever incidence data from a separate systematic review.

**Results:**

Forty-four studies met the inclusion criteria, of which 23 (52.3%) isolated *Salmonella* Typhi at least once. Among studies isolating *Salmonella* Typhi, the median (interquartile range) prevalence and rank order of *Salmonella* Typhi compared to other pathogens isolated in BSI was 8.3% (3.2–37.9%) and 3 (1–6), respectively. The median (interquartile range) S:E ratio was 1.0 (0.4–3.0). With respect to incidence, in Pemba Island, Tanzania, prevalence, rank order, S:E ratio, and incidence was 64.8%, 1, 9.2 and 110 cases per 100 000, respectively, and in Boulkiemdé, Burkina Faso, was 13.3%, 3, 2.3 and 249 cases per 100 000.

**Conclusions:**

We describe considerable variation in place and time for *Salmonella* Typhi prevalence, rank order, and S:E ratio among hospital-based studies of CO-BSI. Data from simultaneous typhoid prevalence and incidence studies are limited. We propose that hospital-based study metrics warrant evaluation for making inference about typhoid incidence and as covariates in typhoid incidence models.

## Introduction

Typhoid fever is a serious systemic infection caused by the organism *Salmonella enterica* subspecies *enterica* serovar Typhi *(Salmonella* Typhi). *Salmonella* Typhi is transmitted predominantly through fecally contaminated food and water [1]. Typhoid fever is an important source of morbidity and mortality globally. It is estimated to cause more than 10 million illnesses and 116 000 deaths [2,3] worldwide, with most illnesses in low-resource areas in Asia and sub-Saharan Africa [4–6]. With the recent prequalification of typhoid conjugate vaccines [7], countries are faced with making decisions about vaccine introduction based on incidence data that are often either scarce, of insufficient quality, or that offer an incomplete picture. Such decisions are complicated by the substantial variation in typhoid incidence not just between regions, but between countries within the same region, and within the same country [8–10].

The reference standard method for estimating the incidence of typhoid fever is prospective, population-based active surveillance in a large cohort, but such studies are costly and time-consuming to implement. Prospective, passive sentinel site surveillance study designs that use healthcare utilisation surveys, called ‘multiplier studies’ [11,12] or ‘hybrid surveillance’ [13,14], make adjustments for under-ascertainment to estimate incidence rates from sentinel site data. These studies are less resource-intensive but yield results that may be susceptible to selection and recall bias, compromise precision, and the type of multipliers implemented are not standardised [8].

Statistical models using historical disease patterns and covariates on the causal pathway of transmission (e.g. water supply, sanitation, and chronic carriers) are an additional avenue for estimating typhoid incidence [15–19]. Surrogates of economic development such as infrastructure (e.g. proportion of road paved), access to improved water and sanitation, prevalence of stunting, and percent of the population living in extreme poverty have also been explored for use in models [5], along with seasonal and environmental factors that may influence typhoid transmission dynamics [20–25].

Covariates for incidence that might be directly related to disease occurrence include metrics from hospital-based studies of community-onset bloodstream infections (CO-BSI), which take into account the prevalence of *Salmonella* Typhi versus that of other BSI, and the rank order of *Salmonella* Typhi among BSIs. To assess the influence of study design and temporal changes, it could be useful to compare the prevalence of *Salmonella* Typhi to the prevalence of non-*Salmonella* organisms, a strategy that has been implemented in epidemiologic studies of pneumococcal disease [26]. We performed an analysis of a systematic review of the prevalence of CO-BSI among hospitalised febrile inpatients with the objective to describe the three hospital-based metrics of *Salmonella* Typhi, to compare them to high-quality primary incidence data from the literature, and to create a resource for future modelling efforts.

## Methods

### Search strategy and selection criteria

The protocol for the systematic review on the prevalence of CO-BSI among febrile hospitalised patients has been published [27] and was registered on PROSPERO on 28 September 2018 (CRD42018109388; Appendix S1). In brief, on 19 September 2018, we searched PubMed, Web of Science and Scopus to identify studies of CO-BSI with no restriction on language, country or date. Keywords used were *fever, bacteremia, septicemia, epidemiology, incidence* and *prevalence*, as well as spelling alternatives and related terms. Prospective studies with consecutive series of hospitalised febrile patients using aerobic blood culture as the reference standard diagnostic test were included.

Two authors screened the titles and abstracts for inclusion. Full-text articles and data abstraction of included articles were independently screened in parallel by two authors with discrepancies resolved by discussion or a separate, third author. Quantitative data abstracted were the number of participants in the study, number of hospitalised participants with BSI, and number and type of each pathogenic organism causing BSI. We subsequently stratified the data of included articles by whether *Salmonella* Typhi was isolated at least once and performed a sub-analysis on these studies. Recognising the importance of negative studies, we also describe the studies from which *Salmonella* Typhi was not isolated.

### Data analysis

Among studies that isolated *Salmonella* Typhi, the number of unique pathogen types was counted by totalling the number of different isolates by species and serovar. Groups of organisms that were not typed or differentiated by the original study, such as ‘non-typhoidal *Salmonella’* or *‘Streptococcus* species,’ were counted as a single type. For example, if a study reported isolating 100 *Salmonella* Typhi, 100 *Escherichia coli*, and 100 ‘non-typhoidal *Salmonella,’* we reported three pathogen types in that study.

We then calculated the prevalence of *Salmonella* Typhi and *E. coli* among pathogens causing BSI and ranked the organisms by proportion of pathogens isolated, where the most frequently isolated organism was ranked first. In studies that also isolated *E. coli*, we compared it to *Salmonella* Typhi by calculating the ratio of *Salmonella* Typhi prevalence to *E. coli* prevalence (S:E ratio). We chose *E. coli* as a comparator organism because of its high prevalence as a cause of bloodstream infection [28,29] and the lack of a vaccine in programmatic use for extraintestinal pathogenic *E. coli.* We did not examine *Staphylococcus aureus* because of the expected low prevalence in our dataset and we ruled out *Streptococcus pneumoniae* and *Haemophilus influenzae* B due to the widespread but incomplete introduction of vaccines for these pathogens. Because not all studies included mycobacterial blood culture in addition to standard aerobic blood culture, mycobacterial isolates were excluded when calculating prevalence, rank, and S:E ratio. Organisms not identified but still attributed as a cause of BSI were also excluded. We reported each hospital-based metric by individual study and the median and interquartile range (IQR 25–75%) of the metrics stratified by United Nations (UN) sub-region [30]. We also documented the UN sub-region and country of negative studies to describe the locations that did not isolate *Salmonella* Typhi and compared the locations on regional maps to studies that isolated *Salmonella* Typhi.

Our previous systematic review reported studies that used population-based surveillance to estimate typhoid fever incidence by location [8]. Due to the observed heterogeneity of typhoid incidence [8–10], we were confident only in comparing hospital-based metrics to typhoid incidence if the studies overlapped by both place and time. Analysis for trends, correlations, and associations were planned but could not be completed due to the lack of overlapping data. We instead describe the number and location of studies from both reviews that overlapped in place and provide a descriptive summary of those that overlap by place and time.

## Results

**Figure 1:**
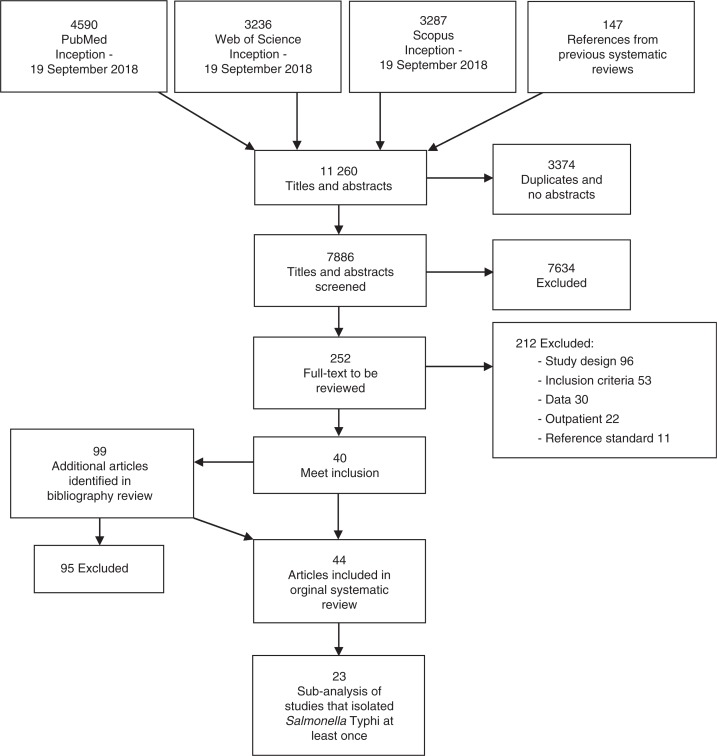
Preferred reporting items for systematic reviews and meta-analyses flow diagram of search strategy and selection of articles that isolated *Salmonella* Typhi among community-onset bloodstream infections, global, 1946–2018.

In our systematic review of the prevalence of CO-BSI among febrile hospitalised patients, we screened 7886 titles and abstracts, of which 7634 were excluded [27]. We then screened the full text of 252 articles, resulting in 44 studies that were included. Among the 44 included studies, 23 (52.3%) studies isolated *Salmonella* Typhi at least once and were eligible for sub-analysis [31–53] (Figure 1).

### Study characteristics

The 23 studies that isolated *Salmonella* Typhi collected data between 1984 and 2014 in 13 countries in Africa (7) and Asia (6) (Table 1). By UN sub-region, 14 (60.9%) studies were done in Eastern Africa, three (13.0%) in South-eastern Asia, two (8.7%) in Southern Asia, and the remaining four (17.4%) in Middle Africa, Northern Africa, Western Africa, and Eastern Asia. There were 23 526 hospitalised febrile participants, of whom 2385 (10.1%) had BSI; the median (IQR) prevalence of BSI was 13.1 (7.0–17.6%).

**Table 1:** Characteristics of 23 studies isolating *Salmonella* Typhi among hospitalised febrile participants by UN sub-regions in Africa and Asia, 1984–2014

UN subregion	Locality, Country [ref]	Inclusion Age	Data collection year(s)	Number of febrile participants	BSI (% of febrile participants)	Count of pathogen types	Three most frequently isolated pathogens (number isolated)
Eastern Africa	Mumias, Kenya [46]	>5 y	1994	229	51 (22.3)	9	*Salmonella* Typhi (24) *Streptococcus pneumoniae* (10) *Salmonella* Enteritidis (6)
	Nairobi, Kenya [36]	3 m–12 y	2001	264	32 (12.1)	10	*Salmonella* Typhimurium (11) *Citrobacter* spp (5) (t). *Staphylococcus aureus* (4) and *Enterococcus* spp (4)
	Blantyre, Malawi [39]	Children (no age provided)	1996–1997	2123	365 (17.2)	17	*Salmonella* Typhimurium (107) *Enterobacter* spp (70) *Streptococcus pneumoniae* (59)
	Blantyre, Malawi [35]	Adults (no age provided)	1997–1998	2789	449 (16.1)	17	*Streptococcus pneumoniae* (137) *Salmonella* Typhimurium (128) *Escherichia coli* (43)
	Blantyre, Malawi [31]	≥14 y	2000	352	69 (19.6)	13	*Salmonella* Typhimurium (28) (t). *Salmonella* Enteritidis (16) and *Streptococcus pneumoniae* (16)
	Lilongwe, Malawi [48]	≥14 y	1998	238	54 (22.7)	13	*Salmonella* Typhimurium (15) Unspecified NTS (9) *Cryptococcus* spp (7)
	Dar es Salaam, Tanzania [43]	≥15 y	1995	517	84 (16.2)	20	*Salmonella* Enteritidis (14) *Staphylococcus aureus* (13) *Escherichia coli* (12)
	Dar es Salaam, Tanzania [34]	0–7 y	2001–2002	1787	127 (7.1)	23	(t). *Escherichia coli* (24) and *Enterococcus* spp (24) *Klebsiella* spp (19)
	Moshi, Tanzania [45]	≥13 y	2007–2008	403	54 (13.4)	12	*Salmonella* Typhi (26) 2(t). *Escherichia coli* (7) and *Streptococcus pneumoniae* (7)
	Moshi, Tanzania [44]	2 m–<13 y	2007–2008	467	16 (3.4)	5	1 *Salmonella* Typhi (6) *Streptococcus pneumoniae* (5) *Escherichia coli* (3)
	Muheza, Tanzania [52]	2 m–13 y	2006–2007	3639	341 (9.4)	8	Unspecified NTS (160) *Streptococcus pneumoniae* (56) *H. influenza* (39)
	Muheza, Tanzania [49]	≥13 y	2007	198	26 (13.1)	9	(t). *Streptococcus pneumoniae* (5) and unspecified NTS (5) (t). *Escherichia coli* (4) and *Streptococcus pyogenes* (4)
	Pemba Island, Tanzania [50]	>2 m	2009–2010	2209	79 (3.6)	5	*Salmonella* Typhi (46) *Streptococcus pneumoniae* (12) (t). *Escherichia coli* (5) and *Staphylococcus aureus* (5)
	Jinja, Uganda [37]	6 m– <60 m	2012	250	45 (18.0)	10	*Staphylococcus aureus* (19) Unspecified NTS (11) *Pseudomonas* spp (5)
Middle Africa	Bangui, Central African Republic [38]	All ages	1999	131	35 (26.7)	8	*Salmonella* Typhimurium (19) *Streptococcus pneumoniae* (7) 3(t). *Salmonella* Typhi (2), *Salmonella* Enteritidis (2), and *Escherichia coli* (2)
Northern Africa	Port Sudan, Sudan [42]	≥12 y	1984	100	22 (22.0)	3	*Salmonella* Typhi (13) *Salmonella* Paratyphi A (5) *Streptococcus pneumoniae* (4)
Western Africa	Boulkiemde, Burkina Faso [47]	2 m–15 y	2013–2014	1339	118 (8.8)	13	*Salmonella* Typhimurium (48) *Salmonella* Enteritidis (17) *Salmonella* Typhi (16)
Eastern Asia	Taipei, Taiwan [53]	≤15 y	NR	300	6 (2.0)	5	*Escherichia coli* (2) 2 Four pathogens tied (1)
Southeastern	Jayapura, Northeastern Asia Papua, Indonesia [41]	All ages	1997–2000	226	34 (15.0)	6	*Salmonella* Typhi (13) *Escherichia coli* (8) *Streptococcus pneumoniae* (6)
	Siem Reap, Cambodia [32]	<16 y	2009–2010	1225	76 (6.2)	13	*Salmonella* Typhi (22) *Streptococcus pneumoniae* (13) *Escherichia coli* (8)
	Multiple, Thailand [40]	>2 y	1991–1993	1137	36 (3.2)	13	*E. coli* (13) (t). *Staphylococcus aureus* (4) and *Enterobacter* spp (4)
Southern Asia	Multiple, India [33]	≥5 y	2011–2012	1564	124 (7.9)	16	*Salmonella* Typhi (44) *Staphylococcus aureus* (24) *Escherichia coli* (11)
	Kathmandu, Nepal [51]	≤12 y	2005–2006	2039	142 (7.0)	19	*Salmonella* Typhi (53) *Streptococcus pneumoniae* (22) *Staphylococcus aureus* (11)

Ref, reference; (t), tied; NR, not reported; BSI, bloodstream infection; NTS, non-typhoidal *Salmonella;* y, years; m, months.

**Figure 2:**
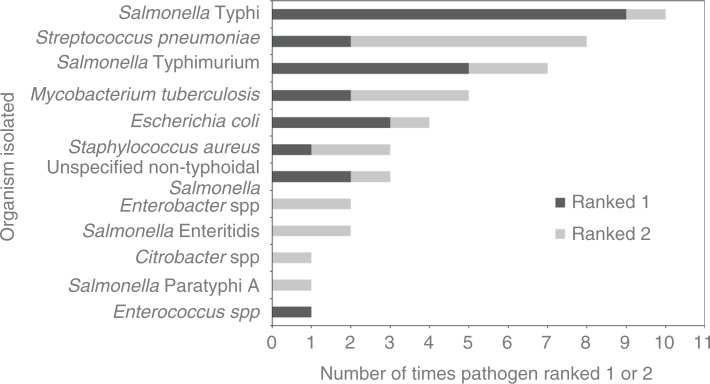
Rank order of isolated pathogens causing BSI, Africa and Asia, 1984–2014.

From participants with BSI, 2413 pathogenic organisms were isolated. The median (IQR) count of pathogen types per study was 12 (8–15). *Salmonella* Typhi was the most frequently isolated organism in nine (39.1%) of the studies, followed by *Salmonella* serovar Typhimurium in six (26.1%), and *E. coli* in three (13.0%) studies (Figure 2).

### Hospital-based metrics

Of 2413 pathogens isolated, 317 (13.1%) were *Salmonella* Typhi. Overall median (IQR) prevalence of *Salmonella* Typhi among pathogens causing BSI was 8.3% (3.2–37.9%); 5.7% (2.7–37.5%) in Africa and 32.7% (19.7–37.8%) in Asia (Table 2). In the UN sub-regions Eastern Africa, South-eastern Asia, and Southern Asia, the median (IQR) prevalence of *Salmonella* Typhi among pathogens was 3.7% (2.3–30.1%), 28.9% (18.6–33.6%), and 37.8% (37.2–38.3), respectively. Overall median (IQR) rank of *Salmonella* Typhi among pathogens causing BSI was 3 (1–6) and was 6 (2–7), 1 (1–3), and 1 (1–1) in Eastern Africa, South-eastern Asia, and Southern Asia, respectively.

**Table 2:** Prevalence and rank order of *Salmonella* Typhi among isolated pathogens causing BSI and *Salmonella* Typhi: E. *coli* ratio, by United Nations sub-region, Africa and Asia, 1984–2014

Locality, Country (last obs year) [ref]	Number of pathogens isolated causing BSI	Proportion of isolates that were *Salmonella* Typhi (%)	*Salmonella* Typhi rank	Proportion of isolates that were *E. coli* (%)	*E. coli* rank	*Salmonella* Typhi: *E. coli* Ratio
*Eastern Africa*						
Pemba Island, Tanzania (2010) [50]	71	64.8	1	7.0	3	9.2
Mumias, Kenya (1994) [46]	52	46.2	1	3.8	6	12.0
Moshi, Tanzania (2008) [45]	58	44.8	1	12.1	2	3.7
Moshi, Tanzania (2008) [44]	16	37.5	1	18.8	3	2.0
Muheza, Tanzania (2007) [49]	25	8.0	5	16.0	3	0.5
Lilongwe, Malawi (1998) [48]	49	6.1	5	6.1	5	1.0
Blantyre, Malawi (1997) [39]	365	4.1	6	0.0	NR	*
Muheza, Tanzania (2007) [52]	341	3.2	7	6.7	5	0.5
Nairobi, Kenya (2001) [36]	32	3.1	6	3.1	6	1.0
Blantyre, Malawi (1998) [35]	450	2.7	8	9.6	3	0.3
Jinja, Uganda (2012) [37]	45	2.2	5	0.0	NR	*
Blantyre, Malawi (2000) [31]	75	1.3	7	5.3	4	0.3
Dar es Salaam, Tanzania (1995) [43]	92	1.1	10	13.0	3	0.1
Dar es Salaam, Tanzania (2002) [34]	155	0.6	16	15.5	1	0.0
Eastern Africa median (IQR)	64.5 (46.0–139.3)	3.7 (2.3–30.1)	6 (2–7)	6.9 (4.2–12.8)	3 (3–5)	0.8 (0.3–2.4)
*Middle Africa*						
Bangui, Central African Republic (1999) [38]	35	5.7	3	5.7	3	1.0
*Northern Africa*						
Port Sudan, Sudan (1984) [42]	22	59.1	1	0.0	NR	_*_
*Western Africa*						
Boulkiemde, Burkina Faso (2014) [47]	120	13.3	3	5.8	5	2.3
Africa median (IQR)	58.0 (35.0–120.0)	5.7 (2.7–37.5)	5 (1–7)	6.1 (3.8–12.1)	3 (3–5)	1.0 (0.4–2.2)
*Eastern Asia*						
Taipei, Taiwan [53]	6	16.7	2	33.3	1	0.5
*South-eastern Asia*						
Siem Reap, Cambodia (2010) [32]	76	28.9	1	10.5	3	2.8
Jayapura, Northeastern Papua, Indonesia (2000) [41]	34	38.2	1	23.5	2	1.6
Multiple, Thailand (1993) [40]	36	8.3	4	36.1	1	0.2
South-eastern Asia median (IQR)	36.0 (35.0–56.0)	28.9 (18.6–33.6)	1 (1–3)	23.5 (17.0–29.8)	2 (2–3)	1.6 (0.9–2.2)
*Southern Asia*						
Kathmandu, Nepal (2006) [51]	145	36.6	1	2.8	10	13.3
Multiple, India (2012) [33]	113	38.9	1	9.7	3	4.0
Southern Asia median (IQR)	129.0 (121.0–137.0)	37.8 (37.2–38.3)	1 (1–1)	6.3 (4.5–8.0)	7 (5–8)	8.7 (6.3–11.0)
Asia median (IQR)	56.0 (34.5–103.8)	32.8 (19.8–37.8)	1 (1–2)	17.0 (9.9–30.9)	3 (1–3)	2.2 (0.8–3.7)
Overall median (IQR)	58.0 (34.5–116.5)	8.2 (3.2–37.9)	3 (1–6)	7.0 (4.6–14.3)	3 (3–5)	1.0 (0.4–3.0)

Ref, reference; BSI, bloodstream infection; NR, not reported; IQR, interquartile range.

* Unable to calculate because demoninator for S:E ratio is zero.

*E. coli* accounted for 186 (7.7%) of 2413 pathogens isolated. Overall median (IQR) prevalence of *E. coli* among pathogens causing BSI was 7.0% (4.6–14.3%); 6.1% (3.8–12.1%) in Africa and 17.0% (9.9–30.9%) in Asia. In the UN sub-regions Eastern Africa, South-eastern Asia, and Southern Asia, the median (IQR) prevalence was 6.9% (4.2–12.8%), 23.5% (17.0–29.8%) and 6.3% (4.5–8.0%), respectively. Overall the median (IQR) rank of *E. coli* among pathogens causing BSI was 3 (3–5) in Eastern Africa, 2 (2–3) in South-eastern Asia and 7 (5–8) in Southern Asia.

The overall median (IQR) S:E ratio was 1.0 (0.5–3.0). Among studies done in Africa, the median (IQR) S:E ratio was 1.0 (0.4–2.2); in Asia it was 2.2 (0.8–3.7). The highest S:E ratio was 13.3 in a study in Kathmandu, Nepal, in 2006, where *Salmonella* Typhi accounted for 53 (36.6%) and *E. coli* for 4 (2.8%) of 145 pathogens isolated [51]. In contrast, the lowest S:E ratio was <0.1 in a study in Dar es Salaam, Tanzania in 2002, where *Salmonella* Typhi accounted for 1 (0.6%) and *E. coli* for 24 (15.5%) of 155 pathogens isolated [34]. Three studies did not isolate *E. coli*, precluding calculation of a S:E ratio [37,39,42].

Our systematic review yielded 21 (47.7%) studies that did not isolate *Salmonella* Typhi, of which seven were done in Africa [54–60] and four in Asia [61–64] (Table 3). The seven studies in Africa were conducted in Kenya, Mozambique, Nigeria, Tanzania, and Uganda. Among these five countries, we identified studies at other locations and times in Kenya, Tanzania, and Uganda that did report isolating *Salmonella* Typhi [34,36,37,43–46,49,50,52]. Five studies in Africa [55,57–60] and one in Asia [61] reported isolating *Salmonella* species but did not specify the species or serovar. Among the 11 studies in Africa and Asia not reporting isolation of *Salmonella* Typhi, all but two reported *E. coli* [54,57]. The median (IQR) prevalence and rank of *E. coli* among these studies were 15.0% (4.6–34.6%) and 2 (1–3), respectively.

**Table 3:** Characteristics of 11 studies not isolating *Salmonella* Typhi among hospitalised febrile participants by UN sub-regions in Africa and Asia, 1984–2014

UN subregion	Locality, Country [ref]	Data collection year(s)	Number of febrile participants	BSI (% of febrile participants)	Count of pathogen types	Three most frequently isolated pathogens (number isolated)
Eastern Africa	Mwanza, Tanzania [59]	2011–2012	317	21 (6.6)	8	*E. coli* (7) *Klebsiella* spp (6) 3(t). *Citrobacter* spp (2) and *Pseudomonas* spp (2)
	Nyanza region, Kenya [54]	2013–2014	148	5 (3.4)	2	Unspecified NTS (4) *Staphylococcus aureus* (1) None
	West Kenya, Kenya [55]	1987–1990	449	58 (12.9)	10	Proteus spp (15) Unspecified *Salmonella* spp (13) *Staphylococcus aureus* (8)
	Maputo, Mozambique [56]	2011–2012	841	63 (7.5)	15	*Staphylococcus aureus* (17) *Escherichia coli* (14) *Salmonella* Typhimurium (9)
	Kampala, Uganda [60]	1997	305	39 (12.8)	11	*Streptococcus pneumoniae* (15) Unspecified *Salmonella* spp (13) *Escherichia coli* (4)
Western Africa	Benin City, Nigeria [57]	1988–1989	642	67 (10.4)	10	*Staphylococcus aureus* (29) Unspecified gram-negative (17) *Alkaligenes faecalis* (10)
	Ibadan, Nigeria [58]	1998	102	39 (38.2)	7	*Escherichia coli* (14) *Staphylococcus aureas* (13) *Klebsiella* spp (4)
Eastern Asia	Tainan, Taiwan [63]	2006–2007	396	60 (15.2)	10	*Escherichia coli* (29) *Klebsiella* spp (13) Unspecified *Streptococcus* spp (7)
	Okinawa, Japan [62]	NR	526	40 (7.6)	7	*Escherichia coli* (13) Unspecified gram-negative (7) (t). *Staphylococcus aureus* (5) and *Klebsiella* spp (5)
South eastern Asia	Bangkok, Thailand [64]	1997	246	119 (48.4)	19	*Cryptococcus neoformans* (31) *Staphylococcus Aureus* (7) *Salmonella* Typhimurium (6)
Southern Asia	Pune, India [61]	2013–2015	1524	59 (3.9)	16	*Acinetobacter* spp (13) *Escherichia coli* (9) (t). *Staphylococcus aureus* (6) and *Enterococcus* spp (6)

Ref, reference; (t), tied; NR, Not reported; BSI, bloodstream infection; NTS, non-typhoidal *Salmonella.*

### Prevalence studies compared to incidence studies

**Figure 3:**
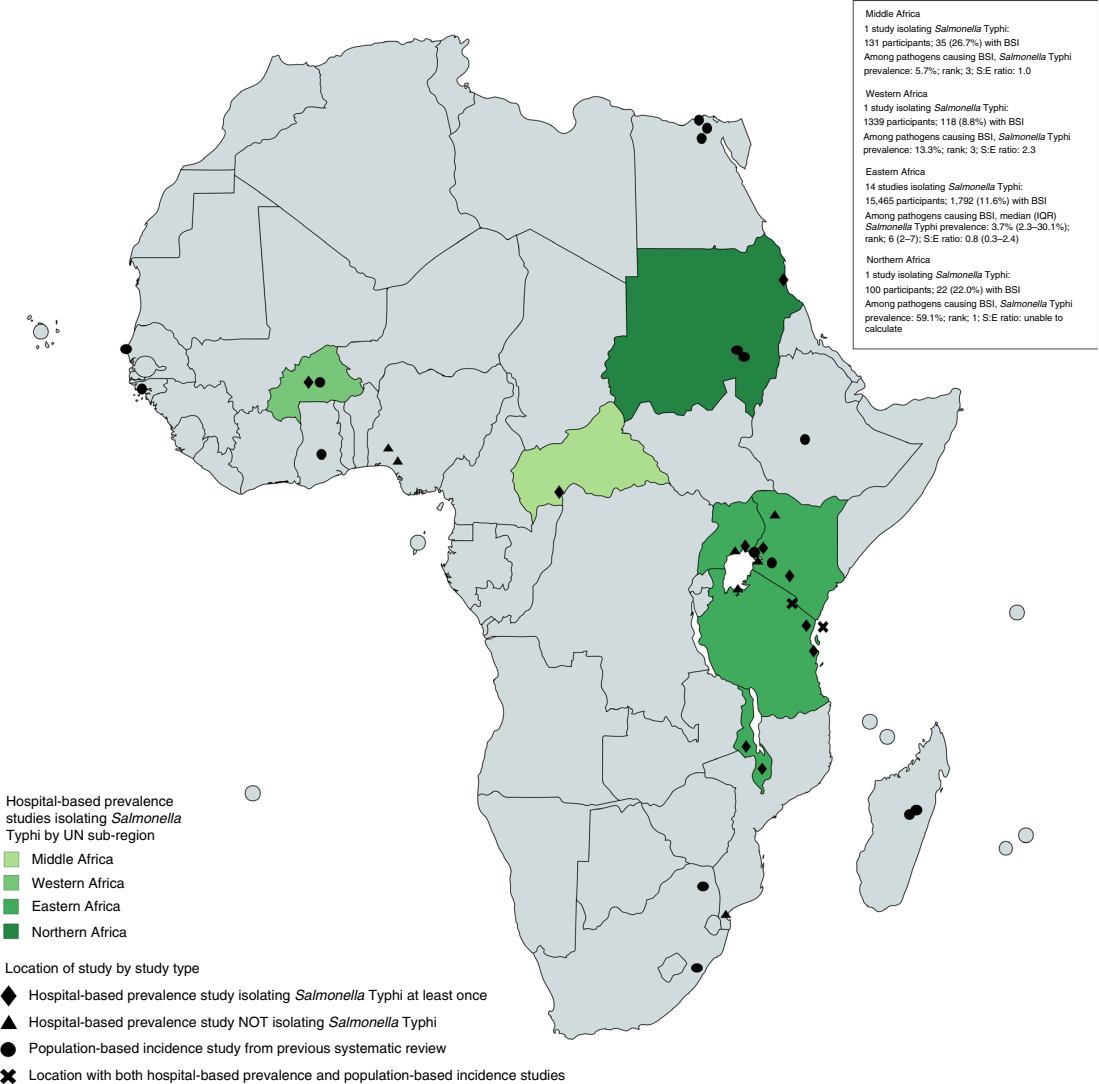
Location of hospital-based prevalence and population-based incidence studies by study type and United Nations sub-regions in Africa [77].

**Figure 4:**
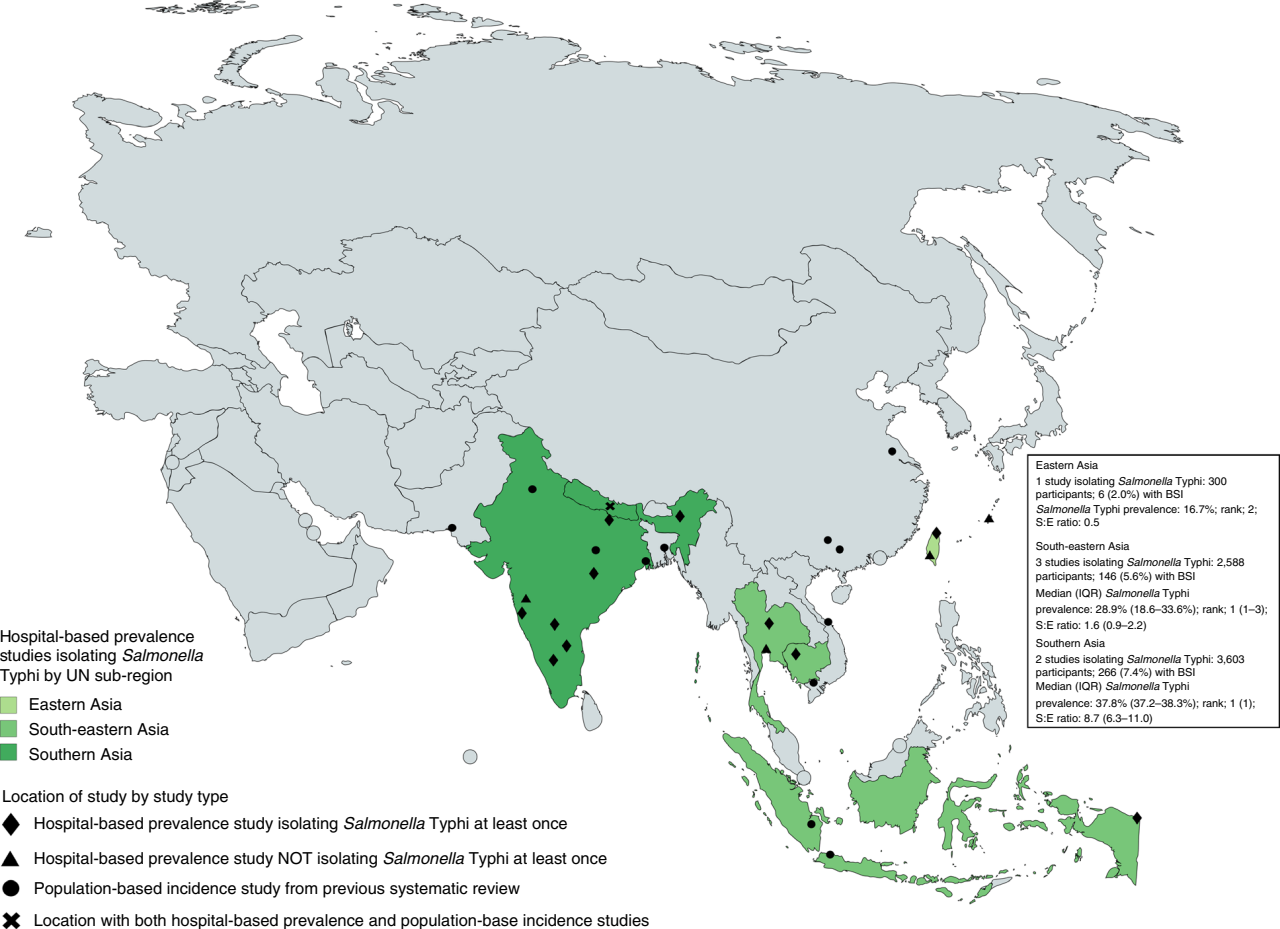
Location of hospital-based prevalence and population-based incidence studies by study type and United Nations sub-regions in Asia [78].

Three hospital-based prevalence studies were done in the same location as a population-based surveillance study of typhoid incidence from our earlier systematic review [8]; two were located in Africa [44,45] (Figure 3) and one in Asia [51] (Figure 4). In Moshi, Tanzania, from 2007 through 2008, typhoid prevalence among pathogens was 37.5%, rank order was 1, and S:E ratio was 2.0 in children aged two months to under 13 years [44]. In rural and urban Moshi in 2011 among children under 15 years, typhoid incidence was 18 and 155 cases per 100 000, respectively. Among adults 13 years and older from 2007 through 2008, typhoid prevalence among pathogens was 44.8%, 1 and 3.7, respectively [45], and typhoid incidence in ages greater than 14 years was 28 and 201 cases per 100 000 in rural and urban Moshi, respectively [65]. In Kathmandu, Nepal from 2005 through 2006 [51], typhoid prevalence among pathogens was 36.6%, rank order was 1, and S: E ratio was 13.3 while incidence was 655 per 100 000 in 1986 [66].

Two locations, Pemba Island, Tanzania in 2010 [50] and Boulkiemdé, Burkina Faso in 2013 [47], had both hospital-based prevalence and a population-based incidence data collected during the same year. Small numbers of concurrent prevalence and incidence studies precluded a statistical examination for an association or trend. In Pemba Island, typhoid prevalence among pathogens was 64.8%, rank order was 1, S:E ratio was 9.2, and typhoid incidence was 110 cases per 100 000. In Boulkiemdé, typhoid prevalence among pathogens was 13.3%, rank order was 3, and S:E ratio was 2.3, and adjusted typhoid incidence was 249 cases per 100 000.

## Discussion

We found that *Salmonella* Typhi prevalence, rank order and prevalence ratio among CO-BSI in hospitalised febrile patients vary substantially in place and time. For example, in three locations in Tanzania, *Salmonella* Typhi prevalence was 37% and the organism ranked first among pathogens isolated in Moshi [44,45]; prevalence was less than 1% and ranked 14 in Dar es Salaam [34] and *Salmonella* Typhi was not isolated in Mwanza [59]. We were only able to directly compare hospital-based prevalence data to studies of population-based incidence in two locations. Because we identified few locations that implement or report on both strategies simultaneously, we were unable to fully investigate the hypothesis that there is a relationship between hospital-based prevalence and population-based incidence.

Based on studies that overlap in place but not time [44,45,51] and also studies not included in our incidence review [10,67–69], it is plausible that areas with high typhoid incidence also observe a high proportion of *Salmonella* Typhi among pathogens isolated from blood cultures. It should be noted that in the only two locations we were able to directly compare the place and time of prevalence to population-based incidence of *Salmonella Typhi*, there was an inverse association, 64.8% prevalence with 110 cases per 100 000 incidence [50] *vs.* 13.3% with a 249 per 100 000 incidence [47]. However, in both of these locations, incidence would be classified as ‘high’ (i.e., greater than 100 cases per 100 000) and *Salmonella* Typhi was among the most frequent pathogens isolated. Blood culture sensitivity [70], proportion of febrile patients seeking hospital care [11,13], and seasonality [21] can lead to varying estimates of incidence [8] and prevalence, limiting the conclusions that can be drawn about the relationship until further investigation, especially given the sample size. We encourage concurrent prevalence and incidence studies to not only examine associations between the two, but also to provide more comprehensive data including on all isolates recovered to assist with informing policy decisions on typhoid control.

Statistical modelling is becoming increasingly important in predicting disease burden in areas where data are lacking [71]. These modelling techniques use what is previously known about a disease and observed data from one location to extrapolate estimates to other locations [72]. For example, epidemiologic studies demonstrate that unsafe water and food, and poor sanitation are associated with increased risk for typhoid fever and are on the causal pathway to infection [73]. Other covariates not directly on the causal pathway, such as population density, wealth distribution, and proportion of roads paved have been used in typhoid modelling [5,15,16]. To our knowledge, covariates that capture the disease state such as those presented in our review, including the hospital-based metrics of prevalence, rank, and ratio compared to other pathogens causing BSI, have not been explored in such models. Generating incidence data by hybrid surveillance requires conducting a representative healthcare utilisation survey in the catchment area of the sentinel surveillance site. Because typhoid prevalence data are considerably easier to collect compared with typhoid incidence data, they may represent an untapped information resource for making inferences about typhoid disease occurence in an area. We call for further data collection and reporting in order to gain further insight into the usefulness of these hospital-based metrics and to test these metrics in typhoid burden models. We anticipate that doing so will deliver more robust and accurate models for estimating typhoid incidence and insights into typhoid occurence outside of the few locations with rigorous incidence studies.

While the majority of studies in the original systematic review isolated *Salmonella* Typhi, a large proportion of studies in our review did not isolate *Salmonella* Typhi. Search strategies for systematic reviews of prevalence and incidence are designed to collect studies in which the pathogen of interest is reported. Because our review was on the prevalence of any CO-BSI, we were able to capture 21 studies that did not isolate *Salmonella* Typhi. It is reasonable to conclude that typhoid fever incidence is unlikely to be substantial in a place where a large prevalence study fails to isolate any *Salmonella* Typhi. Although small studies should be viewed with caution due to their limited power to confirm absence, studies isolating no *Salmonella* Typhi represent important potential sources of information about locations with little or no disease at the time of the study. There are also studies in which participants fit the inclusion criteria for a BSI, but the study only reported on a single pathogenic species, such as *S. pneumoniae* [74,75]. Such studies were not only excluded from our review, but also represent missed opportunities to report the full range of pathogens that were or were not isolated [76].

Our search strategy only included studies on hospitalised participants, where the prevalence of bloodstream infection tends to be considerably higher overall than that found in the outpatient setting. In doing so, we likely missed a proportion of patients that have mild disease, who either do not present to the hospital or are treated as an outpatient or other facilities. We elected not to combine outpatient studies to avoid study location becoming a source of bias but did not attempt to make any adjustments to our analysis to account for underascertainment.

Additionally, we planned to examine the S:E ratio to control the effect of study design on apparent *Salmonella* Typhi prevalence. However, the prevalence and rank of *E. coli* were not stable across our dataset, limiting the usefulness of this metric in our review. An alternative approach would have been to create a composite variable of bloodstream infections other than the target organism for benchmarking. In our view, this approach is confounded by the influence of both other major epidemicprone causes of bloodstream infection such as non-typhoidal *S. enterica* as well as vaccine-preventable infections such as *S. pneumoniae* for which prevalence changes may be driven by vaccine introductions. Given comparators have proven effective for other pathogens [26], we suggest that investigators continue to examine and investigate their performance.

We provide additional evidence through hospital-based prevalence surveillance studies that *Salmonella* Typhi varies in both place and time. Hospital-based studies of CO-BSI may provide a useful window on local disease burden. Continued use of hospital-based prevalence, sentinel site surveillance and active, population-based incidence studies is central to recognising changes in disease dynamics, antimicrobial resistance, and to monitor the impact of vaccine introduction. This review serves as a resource for typhoid disease modellers, and policy makers. We anticipate that hospital-based study metrics warrant consideration as covariates in statistical models and as evidence for decision making for areas beyond those with rigorous studies of typhoid incidence.

## Supporting Information

**Appendix S1** Final search strategy 19 September 2018.
