# Supplementary material for: Using hospital-based studies of community-onset bloodstream infections to make inferences about typhoid fever incidence
Source: Trop Med Int Health. 2019 Dec 21;24(12):1369–83. doi: 10.1111/tmi.13319 (PMC6916262; doi:10.1111/tmi.13319)
Supplement: Supplementary file 1 [file TMIH-2019-TMI-13319-S1.pdf]

Final search strategy 19 September 2018

Pubmed 19 September 18

(incidence[tiab] OR epidemiol*[tiab] OR prevalence[tiab] OR "prevalence"[Mesh] OR etiology[tiab] OR aetiology OR frequency[tiab] OR burden OR "Bacteremia/epidemiology"[MAJR] OR "Fever/etiology"[MeSH Terms])

AND

(febrile[tiab] OR "bloodstream infection" OR "bloodstream infections" OR "blood stream infection" OR "blood stream infections" OR "BSI" OR bacteremia OR bacteraemia OR enteric OR septicemia[tiab] OR septicaemia[tiab])

NOT

(cancer[all] OR malignant[all] OR malignancy[all] OR malignancies[all] OR leukemia[all] OR myeloid[all] OR dental[all] OR periodon*[all] OR tooth[tiab] OR teeth[tiab] OR neutropenia[all] OR neutropaenia[all] OR seizur*[tiab] OR "influenza A"[tiab] OR "influenzae A"[tiab] OR "influenza B"[tiab] OR "influenzae B"[tiab] OR "influenza virus"[tiab] OR "yellow fever"[all] OR "hay fever"[all] OR scarlet[all] OR rheumatic[all] OR norovirus[all] OR arbovirus[all] OR ebola[all] OR sickle[tiab] OR ricket*[tiab] OR hantavirus[all] OR rotavirus[all] OR measles[all] OR chickenpox[all] OR varicella[all] OR animal[tiab] OR pig[tiab] OR horse[tiab] OR foal[tiab] OR equine[tiab] OR hen[tiab] OR chicken[tiab] OR poultry[tiab] OR cow[tiab] OR milk[tiab] OR mouse[tiab] OR mice[tiab] OR heart[tiab] OR surg*[tiab] OR gene[tiab] OR genes[tiab] OR genomic[tiab] OR polymerase[tiab] OR polio[tiab] OR hepatitis[tiab] OR hemorr*[tiab] OR haemorr* OR genetic*[tiab] OR gout[tiab] OR arthritis[tiab] OR cystic[tiab] OR fruit[tiab] OR egg[tiab] OR juice[tiab] OR fresh[tiab] OR assay[tiab] OR plasmid[tiab] OR microarray[tiab] OR array[tiab] OR biomarker[tiab] OR sequencing[tiab] OR sequence[tiab] OR climate[tiab] OR tick[tiab] OR "tick-borne"[tiab] OR "in vitro"[tiab] OR "in vivo"[tiab] OR "in situ"[tiab] OR transplan*[tiab] OR "UTI"[tiab] OR urinary[tiab] OR catheter[tiab] OR "hospital-acquired"[tiab] OR "healthcare-associated"[tiab] OR "healthcare associated"[tiab] OR "hospital-associated"[tiab] OR "HAI"[tiab] OR nosoc*[all] OR "line-associated"[tiab] OR "central line"[tiab] OR "health care-associated"[tiab] OR transfusion[tiab] OR plasma[tiab] OR shock[tiab] OR convul*[all] OR epilep*[all] OR "enteric-coated"[tiab] OR "aspirin"[tiab] OR nutrition*[tiab] OR coronary[tiab] OR "bowel disease"[tiab] OR "crohn's"[tiab] OR pregn*[tiab] OR fetal[tiab] OR "neonatal sepsis"[tiab] OR preterm[tiab] OR "pre-term"[tiab] OR environmental[tiab] OR retrospective[all] OR retrospectively[all] OR "case report"[all] OR "case reports"[all] OR case-control[all]) AND (hasabstract[text] AND Humans[Mesh])

Scopus 19 September 2018

(((TITLE-ABS-KEY (incidence) OR TITLE-ABS-KEY (epidemiology) OR TITLE-ABS-KEY (prevalence) OR TITLE-ABS-KEY (etiology) OR TITLE-ABS-KEY (aetiology) OR TITLE-ABS-KEY (frequency) OR TITLE-ABS-KEY (burden)))

AND

((TITLE-ABS-KEY (febrile) OR TITLE-ABS-KEY ("bloodstream infection") OR TITLE-ABS-KEY ("bloodstream infections") OR TITLE-ABS-KEY ("blood stream infection") OR TITLE-ABS-KEY ("blood stream infections") OR TITLE-ABS-KEY ("BSI") OR TITLE-ABS-KEY (bacteremia) OR TITLE-ABS-KEY (bacteraemia) OR TITLE-ABS-KEY ("enteric") OR TITLE-ABS-KEY (septicemia) OR TITLE-ABS-KEY (septicaemia))))

AND NOT

(((TITLE-ABS-KEY (cancer) OR TITLE-ABS-KEY (malignant) OR TITLE-ABS-KEY (malignancy) OR TITLE-ABS-KEY (malignancies) OR TITLE-ABS-KEY (dental) OR TITLE-ABS-KEY (periodon*) OR TITLE-ABS-KEY (tooth) OR TITLE-ABS-KEY (teeth) OR TITLE-ABS-KEY (neutrop*) OR TITLE-ABS-KEY (seizure*) OR TITLE-ABS-KEY ("yellow fever") OR TITLE-ABS-KEY ("hay fever") OR TITLE-ABS-KEY (*virus) OR TITLE-ABS-KEY (scarlet) OR TITLE-ABS-KEY (rheumatic) OR TITLE-ABS-KEY (ebola) OR TITLE-ABS-KEY (sickle) OR TITLE-ABS-KEY (rickettsia) OR TITLE-ABS-KEY (measles) OR TITLE-ABS-KEY (chickenpox) OR TITLE-ABS-KEY (varicella) OR TITLE-ABS-KEY (pig) OR TITLE-ABS-KEY (horse) OR TITLE-ABS-KEY (foal) OR TITLE-ABS-KEY (equine) OR TITLE-ABS-KEY (hen) OR TITLE-ABS-KEY (chicken) OR TITLE-ABS-KEY (poultry) OR TITLE-ABS-KEY (cow) OR TITLE-ABS-KEY (milk) OR TITLE-ABS-KEY (mouse) OR TITLE-ABS-KEY (mice) OR TITLE-ABS-KEY (heart) OR TITLE-ABS-KEY (surgery) OR TITLE-ABS-KEY (gene) OR TITLE-ABS-KEY (genes) OR TITLE-ABS-KEY (genomic) OR TITLE-ABS-KEY (polymerase) OR TITLE-ABS-KEY ("influenza a") OR TITLE-ABS-KEY ("influenzae a") OR TITLE-ABS-KEY ("influenza b") OR TITLE-ABS-KEY ("influenzae b") OR TITLE-ABS-KEY ("influenza virus") OR TITLE-ABS-KEY ("influenza-like") OR TITLE-ABS-KEY (polio) OR TITLE-ABS-KEY (hepatitis) OR TITLE-ABS-KEY (hemorrhagic) OR TITLE-ABS-KEY (haemorrhagic) OR TITLE-ABS-KEY (genetic) OR TITLE-ABS-KEY (gout) OR TITLE-ABS-KEY (arthritis) OR TITLE-ABS-KEY (cystic) OR TITLE-ABS-KEY (fruit) OR TITLE-ABS-KEY (egg) OR TITLE-ABS-KEY (juice) OR TITLE-ABS-KEY (fresh) OR TITLE-ABS-KEY (assay) OR TITLE-ABS-KEY (plasmid) OR TITLE-ABS-KEY (microarray) OR TITLE-ABS-KEY (array) OR TITLE-ABS-KEY (biomarker) OR TITLE-ABS-KEY (sequencing) OR TITLE-ABS-KEY (sequence) OR TITLE-ABS-KEY (climate) OR TITLE-ABS-KEY (tick) OR TITLE-ABS-KEY ("tick-borne") OR TITLE-ABS-KEY ("in vitro") OR TITLE-ABS-KEY ("in vivo") OR TITLE-ABS-KEY ("in situ") OR TITLE-ABS-KEY (transplantation) OR TITLE-ABS-KEY (transplant) OR TITLE-ABS-KEY ("UTI") OR TITLE-ABS-KEY (urinary) OR TITLE-ABS-KEY (catheter) OR TITLE-ABS-KEY ("hospital-acquired") OR TITLE-ABS-KEY ("healthcare-associated") OR TITLE-ABS-KEY ("healthcare associated") OR TITLE-ABS-KEY ("hospital-associated") OR TITLE-ABS-KEY ("HAI") OR TITLE-ABS-KEY (nosocomial) OR TITLE-ABS-KEY ("line-associated") OR TITLE-ABS-KEY ("central line") OR TITLE-ABS-KEY ("health care-associated") OR TITLE-ABS-KEY (transfusion) OR TITLE-ABS-KEY (plasma) OR TITLE-ABS-KEY (shock) OR TITLE-ABS-KEY (epilepsy) OR TITLE-ABS-KEY (convulsion*) OR TITLE-ABS-KEY ("enteric-coated") OR TITLE-ABS-KEY ("aspirin") OR TITLE-ABS-KEY (nutrition*) OR TITLE-ABS-KEY (coronary) OR TITLE-ABS-KEY ("bowel disease") OR TITLE-ABS-KEY ("crohn's") OR TITLE-ABS-KEY (pregnant)

OR TITLE-ABS-KEY (pregnancy) OR TITLE-ABS-KEY (fetal) OR TITLE-ABS-KEY ("neonatal sepsis") OR TITLE-ABS-KEY (preterm) OR TITLE-ABS-KEY ("pre-term") OR TITLE-ABS-KEY (environmental) OR TITLE-ABS-KEY (retrospective) OR TITLE-ABS-KEY (retrospectively) OR TITLE-ABS-KEY ("case report") OR TITLE-ABS-KEY ("case reports") OR TITLE-ABS-KEY (case-control) OR TITLE-ABS-KEY (animals) OR TITLE-ABS-KEY (animal)))) AND (EXCLUDE (DOCTYPE , "re") OR EXCLUDE (DOCTYPE , "le") OR EXCLUDE (DOCTYPE , "cp") OR EXCLUDE (DOCTYPE , "ed") OR EXCLUDE (DOCTYPE , "no") OR EXCLUDE (DOCTYPE , "sh") OR EXCLUDE (DOCTYPE , "ch") OR EXCLUDE (DOCTYPE , "ip") OR EXCLUDE (DOCTYPE , "er") OR EXCLUDE (DOCTYPE , "bk") OR EXCLUDE (DOCTYPE , "tb")) AND (EXCLUDE (SUBJAREA , "NURS") OR EXCLUDE (SUBJAREA , "AGRI") OR EXCLUDE (SUBJAREA , "PHAR") OR EXCLUDE (SUBJAREA , "NEUR") OR EXCLUDE (SUBJAREA , "VETE") OR EXCLUDE (SUBJAREA , "PSYC") OR EXCLUDE (SUBJAREA , "ENVI") OR EXCLUDE (SUBJAREA , "HEAL") OR EXCLUDE (SUBJAREA , "SOCI") OR EXCLUDE (SUBJAREA , "ENGI") OR EXCLUDE (SUBJAREA , "ARTS") OR EXCLUDE (SUBJAREA , "MULT") OR EXCLUDE (SUBJAREA , "CENG") OR EXCLUDE (SUBJAREA , "CHEM") OR EXCLUDE (SUBJAREA , "EART") OR EXCLUDE (SUBJAREA , "MATH") OR EXCLUDE (SUBJAREA , "PHYS") OR EXCLUDE (SUBJAREA , "COMP") OR EXCLUDE (SUBJAREA , "DENT") OR EXCLUDE (SUBJAREA , "ECON") OR EXCLUDE (SUBJAREA , "MATE") OR EXCLUDE (SUBJAREA , "DECI") OR EXCLUDE (SUBJAREA , "ENER")) AND (EXCLUDE (EXACTKEYWORD , "Nonhuman"))

Web of Science 19 September 2018

1

TS=(incidence) OR TS=(epidemiology) OR TS=(prevalence) OR TS=(etiology) OR TS=(aetiology) OR TS=(frequency) OR TS=(burden)

Indexes=SCI-EXPANDED, ESCI Timespan=All years

2

TS=(febrile) OR TS=("bloodstream infection") OR TS=("bloodstream infections") OR TS=("blood stream infection") OR TS=("blood stream infections") OR TS=("BSI") OR TS=(bacteremia) OR TS=(bacteraemia) OR TS=(enteric) OR TS=(septicemia) OR TS=(septicaemia)
Indexes=SCI-EXPANDED, ESCI Timespan=All years

3

#2 AND #1 NOT TS=(cancer OR malignant OR malignancy OR malignancies OR leukemia OR myeloid OR dental OR periodon* OR tooth OR teeth OR neutropenia OR neutropaenia OR seizure OR seizures OR "influenza A" OR "influenzae A" OR "influenza B" OR "influenzae B" OR "influenza virus" OR "yellow fever" OR "hay fever" OR scarlet OR rheumatic OR norovirus OR Ebola OR sickle OR rickettsia OR hantavirus OR rotavirus OR measles OR chickenpox OR varicella OR pig OR horse OR foal OR equine OR hen OR chicken OR poultry OR cow OR milk OR mouse OR mice OR heart OR surgery OR surgeries OR gene OR genes OR genomic OR polymerase OR polio OR hepatitis OR hemorrhagic OR haemorrhagic OR genetic OR gout OR arthritis OR cystic OR fruit OR egg OR juice OR fresh OR assay OR plasmid OR microarray OR array OR biomarker OR sequencing OR sequence OR climate OR tick OR "tick-borne" OR "in vitro" OR "in vivo" OR "in situ" OR transplant* OR "UTI" OR urinary OR catheter OR "hospital-acquired" OR "healthcare-associated" OR "healthcare associated" OR "hospital-associated" OR "HAI" OR nosoc* OR "line-associated" OR "central line" OR "health care-associated" OR transfusion OR plasma OR shock OR convul* OR epilep* OR "enteric-coated" OR "aspirin" OR nutrition* OR coronary OR "bowel disease" OR "crohn's" OR pregnant OR pregnancy OR fetal OR "neonatal sepsis" OR preterm OR "pre-term" OR environmental OR retrospective OR retrospectively OR "case report" OR "case reports" OR case-control OR animals OR animal)

Refined by: [excluding] DOCUMENT TYPES: (PROCEEDINGS PAPER OR NOTE OR DISCUSSION OR REVIEW OR EDITORIAL MATERIAL OR CORRECTION OR REPRINT OR MEETING ABSTRACT OR LETTER OR BOOK CHAPTER) AND [excluding] WEB OF SCIENCE CATEGORIES: (ENDOCRINOLOGY METABOLISM OR ANESTHESIOLOGY OR CARDIAC CARDIOVASCULAR SYSTEMS OR CHEMISTRY ANALYTICAL OR DENTISTRY ORAL SURGERY MEDICINE OR TRANSPLANTATION OR SURGERY OR OTORHINOLARYNGOLOGY OR SUBSTANCE ABUSE OR VETERINARY SCIENCES OR CLINICAL NEUROLOGY OR ENGINEERING ENVIRONMENTAL OR PHYSICS APPLIED OR PSYCHIATRY OR REPRODUCTIVE BIOLOGY OR NEUROSCIENCES OR DERMATOLOGY OR OBSTETRICS GYNECOLOGY OR UROLOGY NEPHROLOGY OR ECONOMICS OR ENGINEERING ELECTRICAL ELECTRONIC OR ONCOLOGY)
